# The Roles of Contrasting Host Types on the Environmental Abundance of *Anaplasma phagocytophilum*, an Emerging Zoonotic Pathogen

**DOI:** 10.1002/ece3.74308

**Published:** 2026-09-08

**Authors:** William McLellan, Sara Gandy, Jaboury Ghazoul, Fanny Olsthoorn, Livia May, Jude Eze, Mara Rocchi, Hein Sprong, Lucy Gilbert

**Affiliations:** ^1^ School of Biodiversity, One Health and Veterinary Medicine University of Glasgow Glasgow UK; ^2^ Ecosystem Management, Institute of Terrestrial Ecosystems, Department of Environmental Systems Science ETH Zürich Zürich Switzerland; ^3^ School of Veterinary Medicine and Biosciences Scottish Rural College Inverness UK; ^4^ Moredun Research Institute Edinburgh UK; ^5^ National Institute for Public Health and the Environment Bilthoven the Netherlands

**Keywords:** birds, deer, *Ixodes ricinus*, rodents, sheep, ticks

## Abstract

Fundamental knowledge of the role of hosts in shaping vector‐borne pathogen abundance is critical to understanding the ecology of these disease systems; yet the roles can be challenging to tease apart, especially for pathogens vectored by generalist feeders with multiple hosts. In this study, we aimed to quantify the relative contributions of hypothesised pathogen transmission hosts (deer and sheep) and non‐transmission hosts (birds and rodents) to the environmental abundance of *Anaplasma phagocytophilum* (ecotypes I and II) in *Ixodes ricinus* ticks. *Anaplasma phagocytophilum* is a zoonotic pathogen, vectored by *I. ricinus* , that causes tick‐borne fever in livestock and anaplasmosis in humans. We collected data on *A. phagocytophilum* prevalence and environmental hazard (density of infected ticks at 40 sites), sheep presence (at 40 sites) and relative abundance indices of deer (at 40 sites) rodents (at 20 sites) and birds (at 10 sites) in northwest Scotland between 2018 and 2020. As predicted, deer were positively associated with *A. phagocytophilum* prevalence and hazard, indicating their potential role as pathogen transmission hosts. In contrast, bird abundance was negatively associated with *A. phagocytophilum* prevalence, indicating that higher bird densities may divert more ticks away from feeding on transmission hosts, thus reducing *A. phagocytophilum* prevalence. This highlights the importance of including non‐transmission hosts when examining environmental disease risk factors. Sheep presence and rodent abundance index did not significantly influence *A. phagocytophilum* prevalence and hazard at our sites. Our findings could have implications for conservation and disease mitigation as land management practices that reduce deer density and promote bird abundance could reduce *A. phagocytophilum* prevalence and hazard.

## Graphical abstract

In this study, we analysed data collected at 40 sites in the Northwest of Scotland (from 2018 to 2020) to quantify the relative contributions of hypothesised pathogen transmission hosts (deer and sheep) and non‐transmission hosts (birds and rodents) on the environmental abundance of ecotypes I and II of *Anaplasma phagocytophilum* . Our results add to our understanding of the ecology of the *A. phagocytophilum* system, thereby addressing a crucial gap in knowledge, and have implications for conservation and disease mitigation.

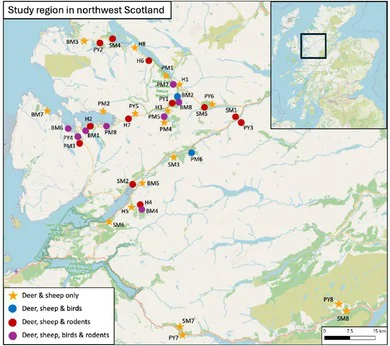

## 1 Introduction

Vector‐borne diseases, which cause 700,000 human deaths worldwide each year (WHO 2020), rely on vectors (often arthropods) to transmit the disease‐causing pathogens between animal or plant hosts (Medlock and Leach 2015; Rulli et al. 2025). The ecologies of vector‐borne pathogens are often highly complex, especially those involving generalist vectors which feed on multiple host species. The relative abundance of different host species will partly determine both the proportion of vectors that are infected with a pathogen (pathogen prevalence) and the density of infected vectors in the environment (environmental hazard of a disease) (Ostfeld and Keesing 2000). Quantifying the role of multiple hosts can be challenging yet crucial for understanding the ecological mechanisms driving vector‐borne pathogen prevalence and hazard. It is particularly important to understand the role of a range of different hosts, as some host species can contribute to higher pathogen prevalence in vector populations while other hosts may contribute to reducing pathogen prevalence (Ostfeld and Keesing 2000). For example, some species of birds and small mammals can transmit *Borrelia burgdorferi* sensu lato (the complex of bacteria which causes Lyme disease in humans), and higher densities of these hosts are associated with higher prevalences of *B. burgdorferi* s.l. within tick populations (De Boer et al. 1993; Dumas et al. 2022; Humair 2002; Tälleklint and Jaenson 1994). In contrast, deer are not able to transmit *B. burgdorferi* s.l. (Kurtenbach et al. 2002; Tälleklint and Jaenson 1994) and higher densities of deer can be associated with a lower proportion of ticks infected with *B. burgdorferi* s.l., because more juvenile ticks feed on deer instead of transmission hosts (Gandy, Kilbride, et al. 2022; Gandy et al. 2021; Rosef, Paulauskas, et al. 2009; Vourc'h et al. 2016). To add further complexity, some types of host can reduce pathogen prevalence while increasing environmental disease hazard. For example, although deer can reduce the proportion of ticks infected with *B. burgdorferi* s.l., their role in feeding large numbers of ticks can result in a neutral or even positive effect on the density of infected ticks (Gandy, Kilbride, et al. 2022; Gandy et al. 2021). This phenomenon of non‐transmission hosts playing an important role in shaping pathogen prevalence and disease hazard is not specific to the *B. burgdorferi* s.l. system and has been shown in other tick‐borne disease systems (Gilbert et al. 2001; Rulli et al. 2025).

*Anaplasma phagocytophilum* is a tick‐borne pathogen complex for which there is a gap in knowledge of how both transmission and non‐transmission hosts shape prevalence and hazard (Sanchez et al. 2016). This is despite *A. phagocytophilum* having substantially higher prevalences in host seeking (“questing”) ticks than *B. burgdorferi* s.l. in some areas (Gandy, Hansford, et al. 2022; Olsthoorn et al. 2021; Rosef, Radzijevskaja, et al. 2009). *Anaplasma phagocytophilum* is an emerging zoonotic pathogenic complex of gram‐negative, intracellular bacteria of both public and veterinary health importance. It causes disease in a wide range of vertebrates (Karshima et al. 2023) including tick‐borne fever in domestic ruminants (Bianchessi et al. 2023; Stuen et al. 2006) and granulocytic anaplasmosis in horses (*Equus ferus caballus*) (Gussmann et al. 2014) and dogs ( *Canis lupus familiaris* ) (El Hamiani Khatat et al. 2021). The pathogen complex is genetically diverse with multiple variants that have been grouped into four (possibly five) distinct “ecotypes” (Bown et al. 2009; Jahfari et al. 2014; Santos et al. 2018; Stuen et al. 2013).

In Europe, ecotypes I and II are vectored primarily by *Ixodes ricinus* ticks (Ixodidae: Ixodidae, Linnaeus; Karshima et al. 2023), the most widespread and abundant tick species in Europe (Gilbert 2021; Gray et al. 2021; Medlock et al. 2013). *Ixodes ricinus* is a generalist feeder which takes blood meals from most terrestrial vertebrate species (Fabri et al. 2022; Gray et al. 2021; Hofmeester et al. 2016), enabling it to transmit pathogens between different host species. *Ixodes ricinus* has three active life stages: larva, nymph and adult, and each stage requires a blood meal before moulting to the next life stage (in the case of larvae or nymphs) or laying eggs (in the case of adult females) (Gray et al. 2021; Herrmann and Gern 2015). Larvae and nymphs commonly feed on small, medium and large sized hosts (Hofmeester et al. 2016; Takumi et al. 2019), whilst adult females rely on larger hosts such as deer or sheep ( *Ovis aries* ; Gray et al. 2021; Herrmann and Gern 2015). Ecotypes III and IV, associated with small mammals and birds respectively (Fabri et al. 2022; Jahfari et al. 2014), are rarely detected in *I. ricinus* ticks (Fabri et al. 2022; Jahfari et al. 2014; Stigum et al. 2019; Takumi et al. 2021) and ecotype V has been so far identified only in *I. ventalloi* ticks from southern Europe (Santos et al. 2018). It therefore appears to be *A. phagocytophilum* ecotypes I and II exclusively that are vectored by *I. ricinus* (Jahfari et al. 2014) and, thus, our study focuses on ecotypes I and II.

*Anaplasma phagocytophilum* has often been reported in the tissues of a range of cervid species and wild and domestic ruminants (Jahfari et al. 2014; Remesar et al. 2022; Stigum et al. 2019; Stuen et al. 2013). In addition, higher *A. phagocytophilum* prevalences in *I. ricinus* have been linked with higher densities of, or presence of, cervids, particularly red deer ( *Cervus elaphus* ), fallow deer ( *Dama dama* ) and roe deer ( *Capreolus capreolus* ) (Fabri et al. 2021; Rosef, Radzijevskaja, et al. 2009; Stigum et al. 2019; Takumi et al. 2021), or domestic ruminants (usually sheep) (Bianchessi et al. 2023; Gandy, Hansford, et al. 2022; Ogden et al. 2002; Perrin 2017). This suggests that cervids and ruminants can be important reservoir hosts. Specifically, ecotype I is the ecotype primarily (but not exclusively) detected in red deer, fallow deer and ruminants, whereas ecotype II is the ecotype primarily (but not exclusively) detected in roe deer (Jahfari et al. 2014; Stigum et al. 2019). Therefore, red and fallow deer as well as other ruminants are likely to be amongst the key transmission hosts for ecotype I, while roe deer are considered to be the main transmission hosts for ecotype II (Fabri et al. 2024; Hamšíková et al. 2019; Stigum et al. 2019). As well as being possible transmission hosts for ecotypes I and II, larger hosts, such as red and roe deer, are often the main drivers of *I. ricinus* tick density (Gandy, Kilbride, et al. 2022; Gilbert et al. 2012; Mysterud et al. 2016; Pacilly et al. 2014). This is because they provide blood meals for large numbers (often over 100 per individual) of all *I. ricinus* tick stages (Kiffner et al. 2010). In particular, deer are amongst the most important hosts that feed adult *I. ricinus* , which can then proceed to reproduce, hence deer act as “tick reproduction hosts” (Gray 1998; Kiffner et al. 2010; Ruiz‐Fons and Gilbert 2010). Hence, as well as augmenting *A. phagocytophilum* prevalence, we might expect deer to also augment the environmental hazard of *A. phagocytophilum* (Fabri et al. 2024, 2022; Takumi et al. 2021, 2019). Similarly, ruminants, such as sheep and cattle can also play a role in maintaining both tick densities and prevalence of *A. phagocytophilum* ecotype I if they have not been treated with acaricide (Laurenson et al. 2007), and so could also play a role in shaping *A. phagocytophilum* hazard.

*Anaplasma phagocytophilum* ecotypes I and II are rarely detected in rodents and have not been detected in birds to date (Jahfari et al. 2014). It is, therefore, assumed that rodents and birds are not transmission hosts. To our knowledge, no previous empirical studies in Europe have analysed data of non‐transmission hosts to assess risk factors for the prevalence or environmental hazard of *A. phagocytophilum* . However, since rodents and birds can be important hosts for larval and nymphal *I. ricinus* (Gray et al. 2021), but are assumed to not infect feeding *I. ricinus* with ecotypes I or II, we might expect higher rodent or bird abundances to be associated with lower prevalence of *A. phagocytophilum* in questing *I. ricinus* (Fabri et al. 2022). This is because, where more larval and nymphal ticks feed on non‐transmission hosts instead of transmission hosts, a higher proportion of the tick population remains uninfected when they emerge as questing nymphs and adults several months later. Indeed, a north American study recently found such a negative association between the prevalence of a particular genotype of *A. phagocytophilum* and a proxy for the abundance of a non‐transmission host (in this case forest fragmentation as a proxy for white‐tailed deer, *Ododoileus virginianus*) (O'Connor et al. 2026).

In this study, we aimed to test the roles of transmission hosts and, importantly, non‐transmission hosts in shaping the prevalence and environmental hazard of *A. phagocytophilum* in questing *I. ricinus* ticks. As *I. ricinus* ticks vector ecotypes I and II, these are the ecotypes inferred when we refer to *A. phagocytophilum* and are the focus of this study. Specifically, we predicted that areas with higher densities of transmission hosts (deer and ruminants) should have higher prevalences and hazards of *A. phagocytophilum* in questing *I. ricinus* ticks (Bianchessi et al. 2023; Gandy, Hansford, et al. 2022; Ogden et al. 2002; Perrin 2017; Rosef, Radzijevskaja, et al. 2009; Stigum et al. 2019). We further predicted that areas with higher densities of non‐transmission hosts (rodents and birds) should have lower prevalences of *A. phagocytophilum* in questing *I. ricinus* ticks. Their effect on hazard, however, is difficult to predict because it also depends on their contribution to the *I. ricinus* population.

We tested these predictions by estimating the prevalence and environmental hazard of *A. phagocytophilum* in questing *I. ricinus* ticks and the presence or relative abundance indices of deer, sheep, birds, and rodents in Wester Ross, northwest Scotland, UK. We surveyed in a range of habitats to ensure a wide variation in host densities between sites.

## 2 Materials and Methods

### 2.1 Survey Design

**FIGURE 1:**
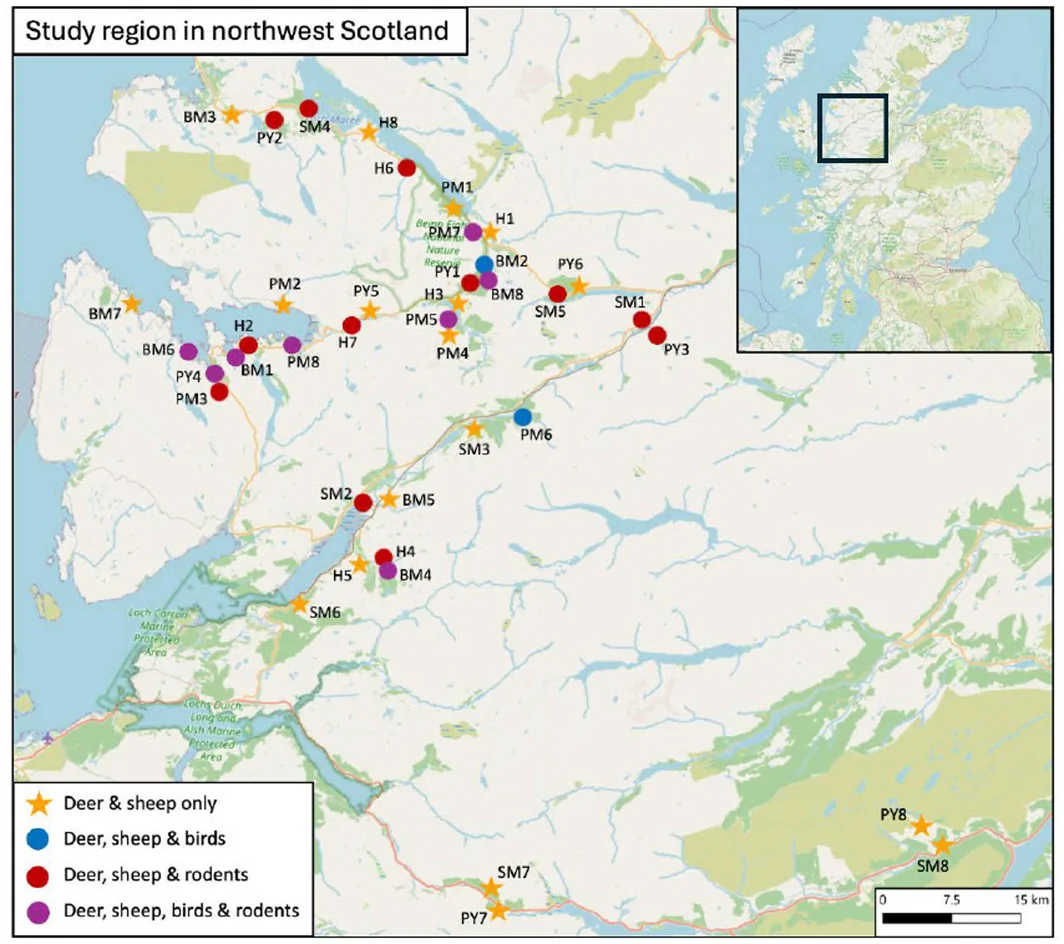
Map of study region within Scotland (inset) showing locations of the 40 study sites. The hosts (deer, sheep, birds, and rodents) surveyed at each site are indicated by colour.

The study region was in Wester Ross in the northwest of Scotland (Figure 1). This is a remote, scenic area, popular with tourists, with hills that rise from sea level on the coastline up to 1000 m elevation. The region comprises a mosaic of forests, rough upland grassland/moorland and montane habitats, with extensively grazed livestock (primarily sheep with small numbers of cattle) and scattered small human settlements. Cervids comprise mainly red deer; roe deer are also present at lower densities. The climate is temperate maritime, characterised by high rainfall (2300 mm per year), cool summers (mean maximum 18.5°C in July) and mild winters (mean minimum 1.3°C in January) (Met Office 2026).

We surveyed 40 sites from 2018 to 2020 (inclusive) for the prevalence and environmental hazard of *A. phagocytophilum* in questing *I. ricinus* ticks and the relative abundance indices of deer (red and roe) and presence of ruminants (sheep; no cattle or goats were present at these sites). Relative abundance indices of rodents and birds were estimated at a subset of 20 and 10 of these sites respectively, due to the resource intensity of the methods involved (Appendix Table A1). The surveyed sites comprised even numbers from each of five habitats representative of the main land cover types in the area: mature birch woodland, mature spruce woodland, mature pine woodland, young pine woodland and open moorland (Appendix Table A1). These were chosen specifically to ensure a wide range of population densities for each of the host types, to better test our predictions about the effect of each host type. The sites encompassed an area of approximately 75 × 75 km, all at low elevation and sharing a similar climate, with adjacent sites being 0.5 to 9 km apart (Figure 1).

### 2.2 *Anaplasma phagocytophilum* Environmental Hazard

*Ixodes ricinus* nymph and adult ticks were collected and their density was estimated using blanket drag transects (Falco and Fish 1992), which involved dragging a 1 × 1 m blanket material over the ground vegetation for 10 m (Gilbert 2010). Twenty transects were conducted for each site visit in a manner that was representative of the range of micro‐habitats present in each site, under the condition that transects were separated by at least 20 m. All 40 sites were surveyed in 2018 over three visits: in spring (May), mid summer (June/July) and late summer (August), to cover the main Scottish tick activity season (MacLeod 1939). In 2019, tick collection and surveys were conducted at a subset of 10 of the 40 sites (for which bird abundance index had been estimated the year before) over two visits (spring and late summer). In 2020, tick collection and surveys were conducted at a subset of 20 of the 40 sites (for which rodent abundance index had been estimated the year before) over one visit (late summer), due to Covid‐19 movement restrictions. All nymphal and adult ticks were counted from each transect and collected into 1.5 mL Eppendorf tubes filled with 70% ethanol for later pathogen analysis.

Environmental hazard is the density of infected tick stages that poses a pathogen hazard to hosts and was thus calculated as the number of infected nymphs and adults per 10 m^2^, that is, per transect. Both nymphs and adults were used because both stages can be infected with *A. phagocytophilum* , posing an “environmental hazard” and both stages bite cervids and ruminants, the transmission hosts that can become infected. Additionally, infection was acquired when these nymphs and adults fed as larvae and nymphs the previous year (or months). These larvae and nymphs feed on all host types (cervids, ruminants, rodents and birds), and our aim was to link these four host categories with pathogen infection in all relevant tick stages that contribute to the transmission cycle and pose a hazard.

As the blanket dragging method can be influenced by vegetation density (Ruiz‐Fons and Gilbert 2010), we recorded ground vegetation height‐density at the beginning, middle, and end of each transect by using a sward stick with coloured tape placed every 5 cm in height. Ground vegetation height‐density was estimated by counting how many bands could not be seen when the stick was placed in the vegetation at arm's length. The average value for each transect was calculated to produce a transect‐level index of ground vegetation height‐density (Gilbert 2010). Air temperature at 15 cm above the ground was recorded using a temperature‐humidity pen (Extech 445580) at the beginning and end of each site visit. The average of those two measurements at the site‐visit level was used so that we could account for temperature‐dependent variation in tick activity (Gilbert et al. 2014). Since both temperature and ground vegetation height‐density index increase throughout our survey season (from May through August), these variables also help account for variation in tick counts over the season.

### 2.3 *Anaplasma phagocytophilum* Prevalence

Nymph and adult *I. ricinus* ticks were washed in distilled water, dried, and individually stored at −20°C until DNA extraction was conducted by boiling individual ticks in 0.7 M ammonium hydroxide as described by Wielinga et al. (2006). Extracted DNA samples were tested for the presence of *A. phagocytophilum* using targets to msp2 (Courtney et al. 2004) genes using a real‐time PCR (qPCR) protocol (Courtney et al. 2004). The qPCRs were carried out on a LightCycler 480 (Roche Diagnostics Nederland B.V., Almere, the Netherlands) in a 20 μL volume, containing iQ Multiplex Powermix, 3 μL of sample, and 0.2 μM of primers and different concentrations of probes. Every PCR plate included positive controls and negative water controls. Samples positive for *A. phagocytophilum* were subjected to a qPCR protocol distinguishing between ecotypes I and II by use of two different probes as described (Gandy, Hansford, et al. 2022). For confirmation of the ecotype specific qPCRs, *A. phagocytophilum‐*positive samples were also sequenced to determine the ecotype, using conventional PCR followed by Sanger sequencing with the GroEL region according to previously published protocols (Jaarsma et al. 2019). *Anaplasma phagocytophilum* prevalence was estimated as the proportion of *I. ricinus* (nymphs plus adults) tested that were infected with either of the *A. phagocytophilum* ecotypes.

Both prevalence and hazard were estimated using both ecotypes together because (i) our deer abundance index was a combination of both red and roe deer; and (ii) both ecotypes are not transmitted by rodents and birds, so we expect areas with more rodents and birds to have lower prevalence of both ecotypes. Hereafter, for the purposes of our study, we use the term *A. phagocytophilum* to mean both ecotypes I and II, unless otherwise specified.

### 2.4 Deer Abundance Index and Sheep Presence

At all 40 sites we counted the number of deer dung piles over the same 10 × 1 m transects over which ticks were surveyed (Gandy, Kilbride, et al. 2022). Deer dung counts were conducted at all sites during the first visit of the year for each site, that is, in May, when ground vegetation is shorter and deer dung detection less prone to error. However, in 2020, all surveys were delayed until August due to Covid‐19 movement restrictions (Appendix Table A1). An index of relative deer abundance was estimated at the site‐year level by averaging the number of deer dung piles per transect for each site for each year. Both red and roe deer were present in the study region. However, due to the difficulty in accurately distinguishing between young red deer and roe deer dung, we combined counts from both species into one deer abundance index.

Sheep presence or absence at each site was established through communication with landowners before visiting each site and confirmed visually during each visit. No other ruminant livestock (cattle or goats) were present at any of our sites. Sheep presence/absence was a site‐level parameter.

### 2.5 Bird Abundance Index

Passerine birds were live‐trapped using mist nets at a subset of 10 sites (five mature Scots pine woodlands and five mature birch woodlands) in 2018. It was not possible to survey birds at more sites as the method is highly resource‐intensive and requires trained and licensed personnel volunteering their time. At each site, two 18 m‐long mist nets were deployed, approximately 200 m apart. The songs of 26 different local bird species were played at both net locations. Mist nets were deployed at each site for four hours in the morning and four hours in the evening of the same day and each net was checked for birds a minimum of once every 30 min. A numbered metal ring (band) was fitted to the leg of each bird which ensured we could identify and discount any individuals that were captured more than once. Mist netting was led by an experienced, qualified, licensed bird handler and mist‐netter under British Trust for Ornithology licence number A4488. For an index of relative abundance of birds to be relevant to the *I. ricinus* ‐ *A. phagocytophilum* system it is important to include only those bird species that carry *I. ricinus* . Therefore, we used the total number of Eurasian chaffinches ( *Fringilla coelebs* ), European robins ( *Erithacus rubecula* ), dunnocks ( *Prunella modularis* ), great tits ( *Parus major* ), and northern wrens ( *Troglodytes troglodytes* ) captured at each site. These were the five species caught that were found to carry ticks in this study and were by far the most frequently caught. As they are ground‐foragers, these are the passerine bird species most implicated as hosts of *I. ricinus* in Scotland (James et al. 2011) and elsewhere in Europe (Keve et al. 2022). While a small number of individuals from other species were caught, we did not include them in analyses as these did not carry ticks at our sites, are not routine ground‐foragers and are not reported to be important hosts to *I. ricinus* in Scotland (James et al. 2011) or elsewhere in Europe (Keve et al. 2022).

### 2.6 Rodent Abundance Index

Rodents were live‐trapped using Longworth traps at a subset of 20 of the 40 sites, limited by the number of personnel and traps available, as it was important to conduct trapping at all sites in a short period of time due to seasonal differences in rodent abundance (Butet et al. 2006). All 20 sites were surveyed for rodents in August/September 2019; and eight of these sites had also been surveyed for rodents in August/September 2018 (Appendix Table A1). Fifty traps per site were laid out on 40 × 40 m grids, where two traps were deployed at every 10 m point on the grid. Traps were deployed over two consecutive nights per site. Each trap was bedded with hay and baited with oats and seeds. The traps had holes in them which would allow shrews (*Sorex* spp.) to escape as per UK small mammal live trapping regulations. Traps were deployed in the evening at 8 p.m., checked and closed the following morning at 8 a.m., and re‐deployed at 8 p.m. All captured mice and voles had their fur clipped to enable identification of re‐trapped individuals. Individual rodents caught more than once, and traps that had failed or been set off by other means, were excluded from estimating the rodent abundance index. The abundance index was the number of newly captured individuals per 100 trap‐nights for each site.

Rodents of three species were captured: bank voles ( *Myodes glareolus* ), field voles ( *Microtus agrestis* ), and wood mice ( *Apodemus sylvaticus* ). Since all three rodent species are considered non‐transmission hosts for *I. ricinus* ‐associated *A. phagocytophilum* (i.e., both ecotypes I and II), we predict they would have similar roles in shaping *A. phagocytophilum* prevalence and hazard. Therefore, the number of captures for each species was combined into a single rodent abundance index for testing our predictions about the role of rodents, as one of the non‐transmission host types, on *A. phagocytophilum* .

There was little evidence of rodent abundance varying consistently between the two rodent survey years (80 per 100 trap‐nights in 2018 and 92 per 100 trap‐nights in 2019); therefore, at sites where rodents were surveyed in both years, an average was taken between the two years to create a more robust estimate. Rodent abundance index was a site‐level variable, with one value per site (as was also the case for birds and sheep).

### 2.7 Statistical Analysis

All statistical analyses were conducted using R (R core Team, version 2022.12.0) and we used Generalised Linear Mixed Effect Models (GLMMs) to test our predictions. For all models, we first tested for potential collinearity between our explanatory variables by calculating the variance inflation factors (VIFs) (Zuur et al. 2009). Collinearity (VIF score > 4) with bird and rodent abundance indices was detected for habitat type. Checks for over‐dispersion and zero‐inflation were carried out on the distributions of the response variables using the zero‐inflation test and the over‐dispersion test in the DHARMa package (Hartig 2022). These compared fitted against simulated residuals and enabled us to specify the appropriate distribution in the models: all hazard data were over‐dispersed beyond the Poisson, so negative binomial distribution was specified for all hazard models.

Because data on rodents and birds were collected at a subset of sites, we used separate models to test for the effects of (i) deer and sheep (using data from all 40 sites) (ii) birds (using data from 10 sites) and (iii) rodents (using data from 20 sites) on *A. phagocytophilum* prevalence and hazard.

#### 2.7.1 Association of Host Types With *A. phagocytophilum* Prevalence in *I. ricinus*

In each of the three models (deer and sheep, birds, rodents), the response variable was the prevalence of *A. phagocytophilum* expressed as the number of nymphs and adults testing positive for *A. phagocytophilum* (ecotypes I or II) over the total number of ticks that were negative, modelled at the site visit level using the lme4 package (Bates et al. 2015). We used binomial GLMMs with logit links. The binomial response used a two‐column matrix of counts (number of positive ticks and number of negative ticks). This formulation is an extension of the binomial framework and is mathematically equivalent to modelling individual‐level binary outcomes, but aggregated at the site visit level. The model therefore estimates the prevalence while appropriately accounting for varying sample sizes of ticks tested across sites. This approach is useful when modelling proportions or prevalence data, as it avoids the loss of information that would occur if site‐level proportions were analysed without incorporating the underlying counts/sample size.

The first model was designed to test the prediction that deer and sheep would augment *A. phagocytophilum* prevalence. Therefore, the key fixed effects of interest were deer abundance index (average number of deer dung piles per transect, at site year level) and sheep presence/absence at the site level (but not rodents or birds, in order to use data from all 40 sites). Habitat (open moorland, young Scots pine, mature Scots pine, mature birch, and mature Sitka spruce) was also included as a fixed effect.

The second and third models were designed to test predictions that birds and rodents, respectively, can reduce *A. phagocytophilum* prevalence. Therefore, the key fixed effects of interest were bird abundance index from the 10 bird survey sites for the bird model and rodent abundance index from the 20 rodent survey sites for the rodent model. Deer index was also included as a fixed effect in both bird and rodent models, as we expected deer to also influence prevalence. The models would not run when sheep presence was included, presumably due to small sample sizes of bird and rodent sites, coupled with very few bird (*n* = 3) and rodent (*n* = 5) sites with sheep present. Habitat was not included as a fixed effect because it covaried with both bird and rodent abundance indices (VIF > 4).

All prevalence models included season of tick survey (categorised as spring, mid summer or late summer) as an additional fixed effect. This was to account for any seasonal effects on prevalence which might operate through seasonal patterns of tick activity and host abundance. All three models included year (2018, 2019, 2020) nested within site as a random effect, since prevalence data from all 3 years of sampling were included in the models and each site was visited across multiple years.

#### 2.7.2 Association of Host Types With Environmental Hazard of *A. phagocytophilum* in *I. ricinus*

To test the effects of (i) deer and sheep, (ii) birds and (iii) rodents on the density of ticks infected with *A. phagocytophilum* (environmental hazard) we used three negative‐binomial GLMMs with log links. A negative‐binomial distribution was specified in the models because tests using the DHARMa package indicated overdispersion beyond the Poisson (Hartig 2022). In each GLMM, the response variable (environmental hazard) was expressed as the number of infected ticks (nymphs and adults) with an offset for the area surveyed, modelled at the site visit level.

As per our prevalence models, host indices were included as fixed effects in each GLMM as follows: the first model included both deer abundance index and sheep presence at all 40 sites, as well as habitat. The second and third models included bird and rodent abundance indices respectively, as well as deer abundance index, as deer are expected to influence both components of hazard: prevalence and density of ticks. As for the prevalence models, the hazard models using the smaller data sets for rodents and birds would not run with sheep presence/absence, so sheep could not be included in the bird and rodent models. Habitat was not included as a fixed effect because it covaried with both bird and rodent abundance indices (VIF > 4).

All hazard models included ground vegetation height‐density index, air temperature and season as fixed effects. The inclusion of ground vegetation height‐density index accounted for its potential effect on the efficiency of the blanket drag method (Ruiz‐Fons and Gilbert 2010). Air temperature affects tick questing activity (Gilbert et al. 2014), and therefore needed to be accounted for. Including season (spring, mid summer, late summer) accounts for seasonal variation in tick activity. These three variables affect the number of ticks counted per 10 m^2^ which is a component of environmental hazard. As with the prevalence models, the random effect was year nested within site.

#### 2.7.3 Model Selection

Model selection was applied to each of the saturated models for both prevalence and hazard using the dredge function from the MuMIn package (Barton 2009) which produces a list of candidate models, with differing fixed effect configurations, ranked according to corrected Akaike information criterion (AICc) where a smaller AICc value indicates a better fit (Brewer et al. 2016). AICc is a modified version of AIC developed for use with smaller sample sizes or when the number of fitted variables is a moderate‐large proportion of the sample size (Hurvich and Tsai 1989), as is the case in our study.

Models with an AICc score within two points (ΔAICc < 2) of each other are generally considered to be of similarly good fit (Mazerolle 2006; Fabozzi 2014). Therefore, the final selected model was not necessarily the model with the lowest AICc score. If a model had an AICc within 2 points of the best model (i.e., lowest AICc), we selected the model which retained the focal host variable (Fabozzi 2014; Mazerolle 2006). Diagnostics from all final selected models were checked using the DHARMa package (comparing fitted and simulated residuals using QQ plots).

## 3 Results

Over the three years of data collection (2018–2020), 9365 *I. ricinus* were collected from the 40 sites: 8867 nymphs and 498 *I. ricinus* adults. The overall average infection prevalence of *A. phagocytophilum* was 5.3% [95% CI = 4.8–5.8] (496/9365) (nymphs and adults). The environmental hazard of *A. phagocytophilum* per site visit was, on average, 11.2 infected *I. ricinus* (nymphs and adults) per km^2^ (SD = 21.6; range = 0–111.3). Out of the 496 ticks that tested positive for *A. phagocytophilum*, 346 could be identified to ecotype: 86% (297/346) were ecotype I, and 14% (49/346) were ecotype II.

The mean deer abundance index was 0.9 deer dung piles per 10 m^2^ (SD = 1.2; range = 0–5.8) and sheep were present at 20% (8/40) of sites. The total number of individual birds (from the five species retained: Eurasian chaffinches, Eurasian wrens, European robins, dunnocks, and great tits) counted in mist‐nets was 77 over the 10 sites where birds were surveyed, and the mean bird abundance index per site was 4.2 (SD = 3.5; range = 0–10). The total number of individual rodents captured (combining wood mice, bank voles and field voles) was 240 across the 20 sites where rodents were surveyed, and the mean rodent index per site was 5.9 per 100 trap‐nights (SD = 4.2; range = 0–13.6) (Table 1).

**TABLE 1:** Summary data showing the mean values for each site of *A. phagocytophilum* prevalence and hazard, the bird and rodent indices and the mean deer abundance index for each site.

Site	*A. phagocytophilum* prevalence per visit (%)	*A. phagocytophilum* hazard per visit (per km^2^)	Deer abundance index per year	Bird abundance index	Rodent abundance index
BM1	5.5	59.0	0.18	7	13.55
BM2	3.6	2.9	0.10	9	
BM3	2.4	12.1	0.75		
BM4	0.5	2.5	0.02	7	8.41
BM5	0.0	0.0	0.00		
BM6	2.1	10.7	0.18	10	8.56
BM7	1.7	1.7	0.20		
BM8	4.2	9.0	0.28	4	3.56
H1	0.0	0.0	0.15		
H2	2.1	0.8	0.08		0.00
H3	4.8	1.5	0.30		
H4	3.5	35.7	1.14		5.49
H5	22.1	45.2	1.90		
H6	0.0	0.0	0.25		5.10
H7	1.3	0.5	0.26		1.04
H8	9.6	2.4	0.50		
PM1	12.5	4.7	0.60		
PM2	1.2	0.4	0.55		
PM3	6.3	33.5	0.97	1	8.80
PM4	7.3	9.4	3.07	1	4.18
PM5	0.0	0.0	0.05		
PM6	11.4	35.1	0.54	0	
PM7	4.0	4.8	0.09	2	12.75
PM8	5.2	21.9	1.00	1	6.93
PY1	3.5	1.7	0.79		4.26
PY2	2.9	2.1	1.79		1.05
PY3	9.3	0.6	0.19		1.08
PY4	10.0	3.1	0.28		1.04
PY5	0.0	0.0	1.95		
PY6	10.6	8.3	4.90		
PY7	10.0	2.2	3.15		
PY8	5.1	3.1	1.85		
SM1	11.0	16.9	2.00		7.37
SM2	9.5	32.9	5.06		2.04
SM3	1.1	1.6	0.25		
SM4	0.0	0.0	0.11		10.31
SM5	4.8	1.0	0.83		2.02
SM6	3.6	4.6	0.25		
SM7	0.0	0.0	0.20		
SM8	0.0	0.0	0.10		

*Note:* BM indicates mature birch, PM is mature pine, PY is young pine, SM is mature spruce and H indicates upland moorland.

### 3.1 Association of Deer and Sheep With *A. phagocytophilum* Prevalence

**FIGURE 2:**
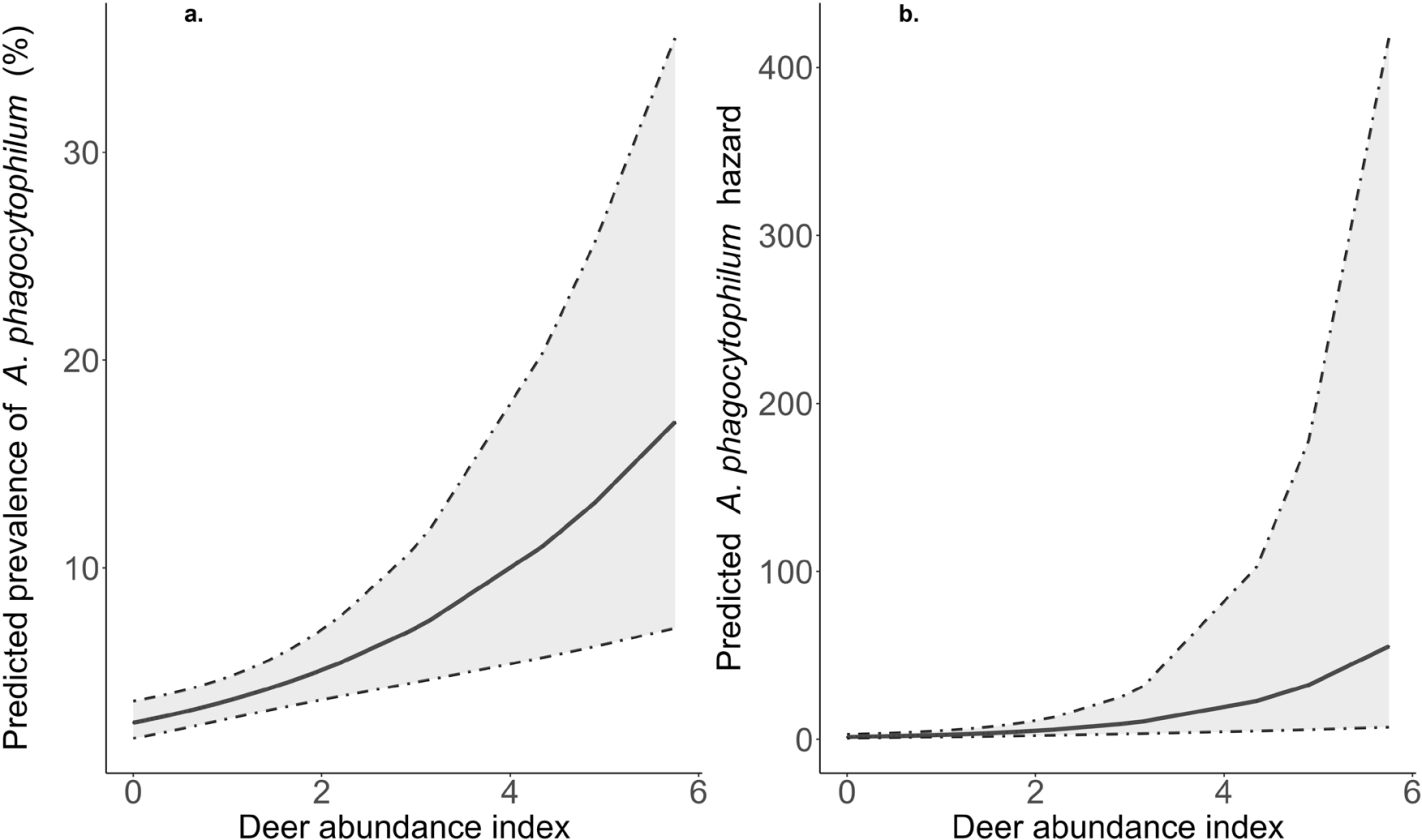
Output model predictions of the effect of deer (roe and red) abundance index (number of deer dung piles 10 per m^2^) on (a) infection prevalence of *Ixodes ricinus* ticks (nymphs and adults) with *Anaplasma phagocytophilum* (%); and (b) environmental hazard of *A. phagocytophilum* (density of infected *I. ricinus* nymphs and adults per km^2^) at 40 sites. The solid lines represent the predicted mean values, and the shaded areas display the 95% confidence intervals.

**FIGURE 3:**
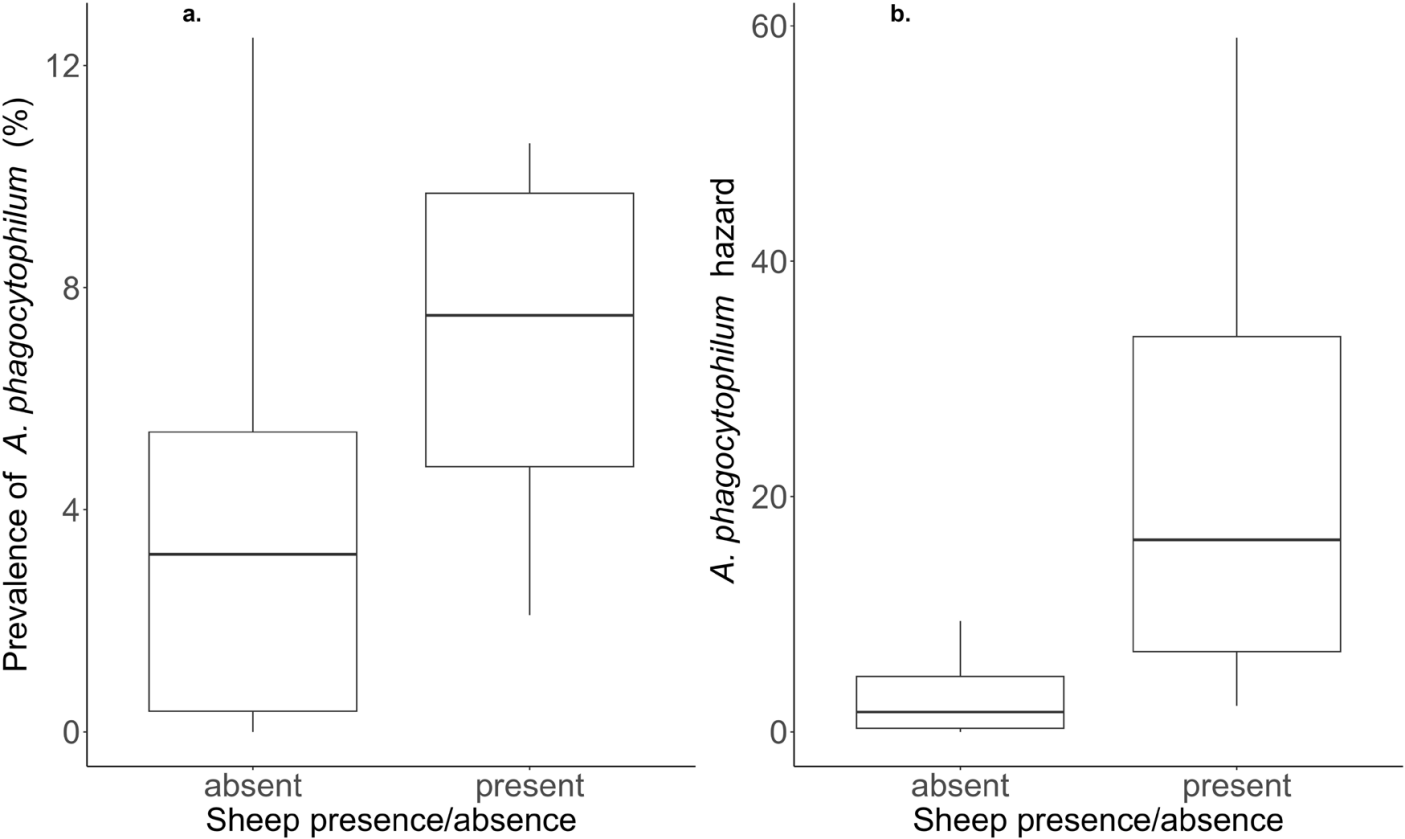
Relationship between sheep presence/absence at the site level and (a) infection prevalence of *Ixodes ricinus* ticks (nymphs and adults) with *Anaplasma phagocytophilum* (%); and (b) environmental hazard of *A. phagocytophilum* (density of infected *I. ricinus* nymphs and adults per km^2^) at 40 sites. Unadjusted raw data are shown. The white boxes depict the interquartile range of prevalence values and the solid horizontal dark line within them represents the mean. The solid lines represent the predicted mean values, and the shaded areas display the 95% confidence intervals.

Deer abundance index was strongly positively correlated with *A. phagocytophilum* prevalence (*p* < 0.001; estimate = 0.36; Figure 2a; Table 2). The mean infection prevalence for *A. phagocytophilum* was, on average, 1.6 times higher in sites where sheep were present compared to where sheep were absent (Figure 3a); however, sheep presence was not retained in the selected model (Table 2). Habitat and season were also not retained in the selected model (Table 2).

**TABLE 2:** Output from the generalised linear mixed effects models showing the effects of deer, birds and rodents on infection prevalence of *A. phagocytophilum* in questing *Ixodes ricinus* nymphal and adult ticks.

Selected focal model	Estimates (logit)	Std error	*p*	*z*‐value	Δ AICc
Deer and sheep (40 sites)					
Intercept	−3.64	0.19	< 0.001	−18.97	
Deer abundance index	0.36	0.10	< 0.001	3.14	9.2
Sheep presence	Excluded from model				
Habitat	Excluded from model				
Season	Excluded from model				
Birds (10 sites)					
Intercept	−2.63	0.23	< 0.001	−11.22	
Bird abundance index	−0.14	0.04	0.001	−3.22	4.8
Deer abundance index	Excluded from model				
Season	Excluded from model				
Rodents (20 sites)					
Intercept	−3.69	0.22	< 0.001	−17.15	
Rodent abundance index	Excluded from model				
Deer abundance index	0.35	0.12	0.003	2.97	5.4
Season	Excluded from model				

*Note:* Delta AICc is the change in AICc if the variable is removed (a negative value denotes a decrease in AICc score, indicating a better model fit; positive values indicate a worse fit). A variable was removed from the model if removing it caused an increase in AICc score of more than two. All variables initially entered into the global model are listed; those that were excluded during model selection are indicated by “Excluded from model”.

### 3.2 Association of Birds With *A. phagocytophilum* Prevalence

**FIGURE 4:**
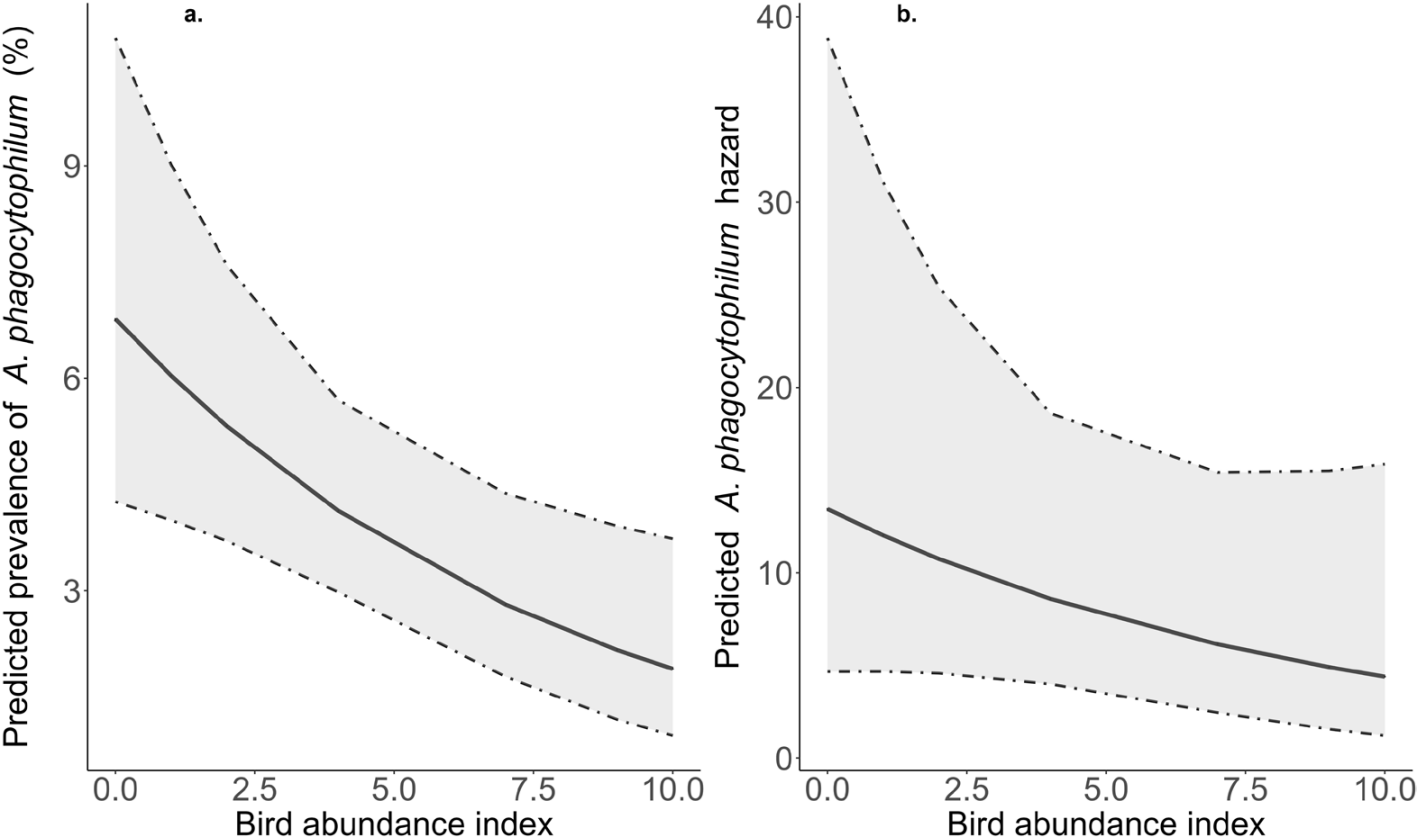
Output model predictions of the effect of bird abundance index (number of birds captured per site) on (a) infection prevalence of *Ixodes ricinus* ticks (nymphs and adults) with *Anaplasma phagocytophilum* (%); and (b) environmental hazard of *A. phagocytophilum* (density of infected *I. ricinus* nymphs and adults per km^2^) at 10 sites. The solid lines represent the predicted mean values, and the shaded areas display the 95% confidence intervals.

*Anaplasma phagocytophilum* prevalence was negatively correlated with bird abundance index (*p* = 0.008; estimate = −0.14; Figure 4a; Table 2). Deer abundance index and season were not retained.

### 3.3 Association of Rodents With *A. phagocytophilum* Prevalence

**FIGURE 5:**
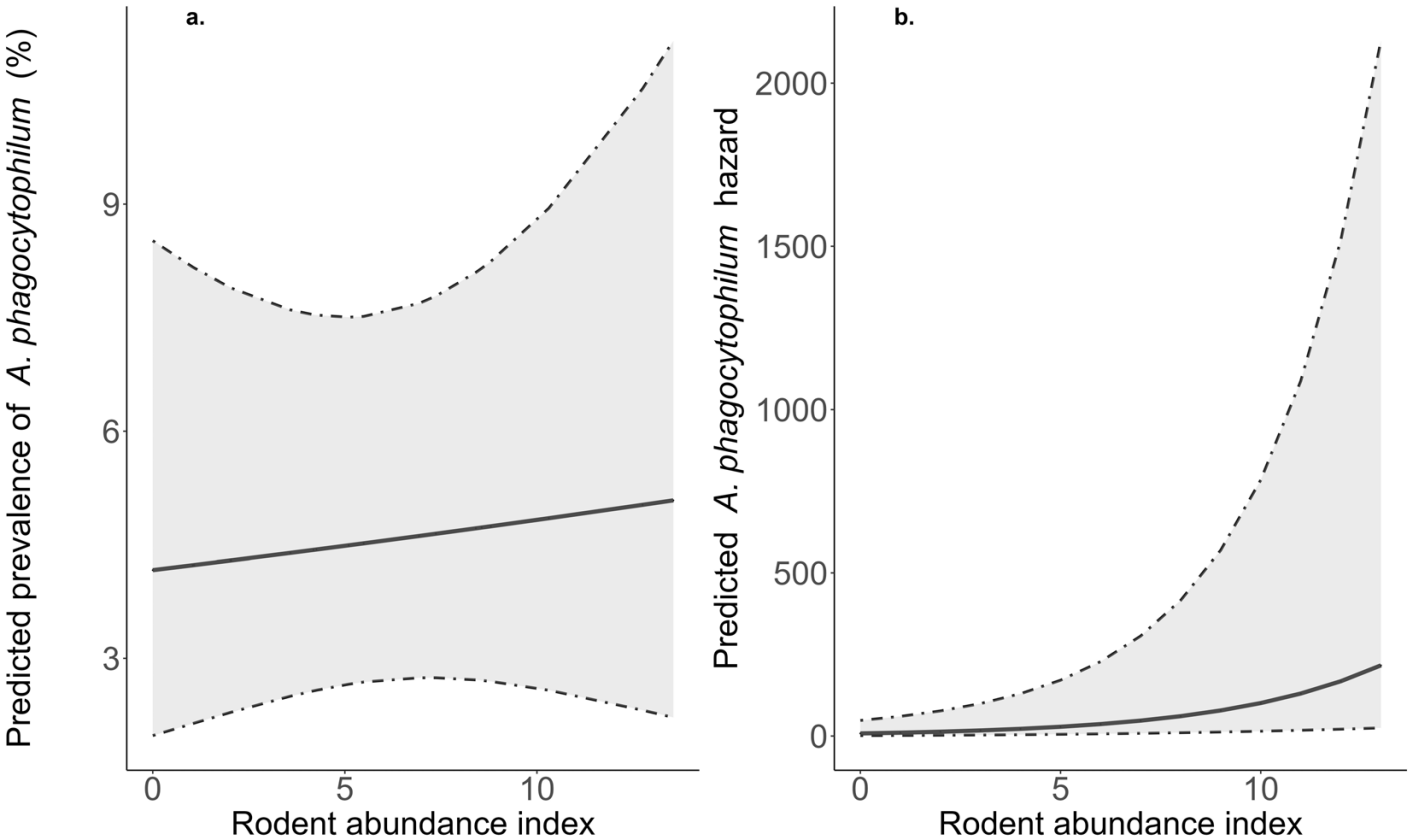
Output model predictions of the effect of rodent abundance index (newly captured rodents trapped/100 trap‐nights per site) on (a) infection prevalence of *Ixodes ricinus* ticks (nymphs and adults) with *Anaplasma phagocytophilum* (%); and (b) environmental hazard of *A. phagocytophilum* (density of infected *I. ricinus* nymphs and adults per km^2^) at 20 sites. The solid lines represent the predicted mean values, and the shaded areas display the 95% confidence intervals. The solid lines represent the predicted mean values, and the shaded areas display the 95% confidence intervals. Note that rodents were not retained in the final selected model, so these predicted values were gained from the (not selected) model with rodents included.

Rodent abundance index was not retained in the model (Figure 5a; Table 2). Deer abundance index was positively associated with prevalence (*p* = 0.004; estimate = 0.35; Table 2). Season was not retained.

### 3.4 Association of Deer and Sheep With *A. phagocytophilum* Hazard

Deer abundance index was strongly positively correlated with the environmental hazard of *A. phagocytophilum* (*p* = 0.002; estimate = 0.63; Figure 2b; Table 3), while spring had the highest predicted *A. phagocytophilum* hazard (Table 3). Sites with sheep had 3.5 times the hazard of sites without sheep (Figure 3b), but sheep presence was not retained in the selected model (Table 3). Habitat, temperature, and ground vegetation height‐density index were also not retained in the selected model.

### 3.5 Association of Birds With *A. phagocytophilum* Hazard

There was no statistically significant association between *A. phagocytophilum* hazard and bird abundance, although birds were retained in the model as including them did not increase AICc by more than 2 (*p* = 0.22; estimate = −0.11; Figure 4b; Table 3). Temperature was negatively correlated with *A. phagocytophilum* hazard and spring was predicted to be the time of year with the highest hazard (Table 3). Ground vegetation height‐density index and deer were not retained in the selected model (Table 3).

### 3.6 Association of Rodents With *A. phagocytophilum* Hazard

There was a positive association between the environmental hazard of *A. phagocytophilum* and rodent abundance index (*p* = 0.02; estimate = 0.24; Figure 5b; Table 3) and with deer abundance index (*p* = 0.005; estimate = 0.83; Table 3). Spring was predicted to have the highest hazard (Table 3). Ground vegetation height‐density index and temperature were not retained (Table 3).

**TABLE 3:** Output from the generalised linear mixed effects models showing the effects of deer, birds and rodents on the environmental hazard of *A. phagocytophilum* (density of infected *Ixodes ricinus* nymphal and adult ticks).

Selected models	Estimates (log)	Std error	*p*	*z*‐value	Δ AICc
Deer and sheep (40 sites)					
Intercept	−5.90	0.37	< 0.001	−16.08	
Deer abundance index	0.63	0.20	0.002	3.11	6.9
Ground vegetation height‐density index	Excluded from model				
Sheep presence	Excluded from model				
Temperature	Excluded from model				
Habitat	Excluded from model				
Season					19.3
Mid summer	−1.01	0.29	< 0.001	−3.53	
Late summer	−1.45	0.26	< 0.001	−5.57	
Birds (10 sites)					
Intercept	−2.35	0.94	0.01	−2.47	
Bird abundance index	−0.11	0.09	0.22	−1.22	−1.3
Temperature	−0.08	0.04	0.05	−1.94	1.0
Ground vegetation height‐density index	Excluded from model				
Deer abundance index	Excluded from model				
Season					9.1
Mid summer	−0.52	0.36	0.14	−1.46	
Late summer	−1.22	0.28	< 0.001	−4.42	
Rodents (20 sites)					
Intercept	−7.20	0.59	< 0.001	−12.20	
Rodent abundance index	0.24	0.06	< 0.001	3.63	7.8
Ground vegetation height‐density index	Excluded from model				
Deer abundance index	0.83	0.21	< 0.001	3.93	11.8
Season					18.1
Mid summer	−0.84	0.29	0.003	−2.94	
Late summer	−1.24	0.25	< 0.001	−5.04	
Temperature	Excluded from model				

*Note:* ΔAICc is the change in AICc if the variable is removed (a negative value denotes a decrease in AICc score indicating a better model fit; positive values indicate a worse fit). A variable was removed from the model if removing it caused an increase in AICc score of more than two. All variables initially entered into the global model are listed; those that were excluded during model selection are indicated by “Excluded from model”.

## 4 Discussion

In this study, we aimed to test the effect of two hypothesised types of transmission hosts, deer and sheep, and two hypothesised types of non‐transmission hosts, birds and rodents, on the prevalence and hazard of *A. phagocytophilum* in questing *I. ricinus* ticks. As predicted, deer abundance index was positively associated with both the prevalence and hazard of *A. phagocytophilum* . Deer have previously been suspected to be transmission hosts for *A. phagocytophilum* (Hamšíková et al. 2019; Rosef, Radzijevskaja, et al. 2009; Stigum et al. 2019; Stuen et al. 2013), and are well known to be amongst the most important hosts driving *I. ricinus* populations (Gandy, Kilbride, et al. 2022; Gilbert et al. 2012; Gray et al. 2021; Gray 1998; Mysterud et al. 2016; Pacilly et al. 2014). Our results are consistent with a study from the Netherlands, which found that the density of *I. ricinus* nymphs infected with *A. phagocytophilum* was higher in areas where the relative availability of red deer, fallow deer, and roe deer (estimated based on camera and live trapping data) were also high (Takumi et al. 2021). Of direct importance to livestock health, deer presence has also been shown to influence *A. phagocytophilum* infection in cattle (Rousseau et al. 2021). The results of our analyses are consistent with these previous studies and provide further evidence that deer can augment the prevalence and hazard of *A. phagocytophilum* ecotypes in *I. ricinus* ticks.

Our average *A. phagocytophilum* prevalence was 5.3% over all our 40 sites in northwest Scotland, which is higher than in northern England (4.7%) and much higher than in southern England (1.8%) (Gandy, Hansford, et al. 2022). This might suggest there could be a general trend of increasing prevalence from south to north in the UK, perhaps reflecting higher sheep and deer densities further north. At our 40 sites, 86% of successfully ecotyped *A. phagocytophilum* samples were ecotype I while 14% were ecotype II. These values are similar to a study across England and Wales that found that positive samples comprised 86.8% ecotype I and 13.2% ecotype II (Gandy, Hansford, et al. 2022), suggesting these ecotype ratios may be typical across the UK. In our study region of Scotland, this likely reflects the greater abundance of sheep and red deer relative to roe deer, since red deer (and sheep) are associated mostly with ecotype I (Fabri et al. 2022, 2021; Jahfari et al. 2014; Rosef, Radzijevskaja, et al. 2009; Stigum et al. 2019) while roe deer are associated mostly with ecotype II (Hamšíková et al. 2019; Jahfari et al. 2014; Stigum et al. 2019). However, due to the difficulty in confidently distinguishing the dung of young red deer from the dung of adult roe deer, our deer abundance index was a combination of roe and red deer dung counts. It would be interesting to investigate the differential roles of red and roe deer separately where they co‐occur by strategically selecting sites that encompass a range of red and roe deer density ratios and statistically analysing ecotype I separately from ecotype II. We might expect that areas with higher roe deer abundance, while having higher prevalence of ecotype II, might have lower ecotype I prevalence if roe deer have poor transmission competency for ecotype I (Fabri et al. 2024). This would require a reliable alternative method of assessing relative abundance to avoid the issues associated with dung identification.

Since ruminants are considered transmission hosts for *A. phagocytophilum* (primarily ecotype I), we predicted that sheep presence at our sites would have a positive influence on *A. phagocytophilum* in *I. ricinus* ticks. Previous research have demonstrated positive associations between sheep presence and *A. phagocytophilum* prevalence (Bianchessi et al. 2023; Gandy, Hansford, et al. 2022; Ogden et al. 2002; Perrin 2017). Similarly, we found the mean infection prevalence and hazard for *A. phagocytophilum* were 1.6 and 3.5 times higher (respectively) in the sites where sheep were present compared to where sheep were absent. However, sheep presence was not retained in our selected models, likely because sheep were only present in eight of our 40 survey sites. In addition, presence/absence data do not allow for analysis of a quantitative range in sheep densities. Furthermore, the role of sheep in shaping *I. ricinus* tick and tick‐borne pathogen abundance can be strongly influenced by the application of acaricides (Laurenson et al. 2007). To better understand the role of sheep in influencing the ecology of *A. phagocytophilum* , analysis of *A. phagocytophilum* prevalence and hazard across a range of sheep densities would provide deeper insight. This would be particularly relevant for informing potential mitigation strategies for tick‐borne fever in sheep, such as managing stocking densities, or targeting acaricide treatments depending on stocking density‐related risk.

As predicted, we found a negative correlation between bird abundance and the prevalence of *A. phagocytophilum* in *I. ricinus*, an empirical association that has not been tested previously, to the best of our knowledge. Since previous studies have not found *A. phagocytophilum* ecotypes I and II in bird tissue samples or ticks collected from birds (Jahfari et al. 2014), it is likely that birds do not support transmission of *A. phagocytophilum* to *I. ricinus* (i.e., ecotypes I or II). Therefore, where there are higher densities of birds, a higher proportion of immature *I. ricinus* are likely to feed on birds instead of on pathogen transmission hosts, thus resulting in lower pathogen prevalence in the tick population. This effect has been observed in relation to high densities of deer reducing *B. burgdorferi* s.l. prevalence in questing *I. ricinus* ticks (Gandy, Kilbride, et al. 2022; Gandy et al. 2021; Rosef, Paulauskas, et al. 2009; Vourc'h et al. 2016), because deer can feed large number of ticks (Gray et al. 2021; Kiffner et al. 2010; Mysterud et al. 2014) but do not transmit *B. burgdorferi* s.l. (Tälleklint and Jaenson 1994). In addition, a north American study found a similar dilution effect of white‐tailed deer (using forest fragmentation as a proxy) on *A. phagocytophilum* prevalence genotypes that are not transmitted by deer (O'Connor et al. 2026). Our finding that birds may reduce *A. phagocytophilum* prevalence further demonstrates the importance of including non‐transmission hosts in analyses of pathogen ecology, even when individuals of those host species do not tend to carry large tick burdens (James et al. 2011).

One potential alternative explanation for areas with fewer birds having higher *A. phagocytophilum* prevalence could be if deer (that augment prevalence) are a confounding factor. We found that higher densities of deer were associated with higher *A. phagocytophilum* prevalence. Higher deer densities could potentially correlate with lower bird densities because of grazing pressure adversely impacting bird habitat (Crystal‐Ornelas et al. 2021). Therefore, a negative association between bird abundance index and prevalence could occur if sites with higher deer abundance have lower bird abundance. However, this is unlikely to be the case in our study because we included deer in all the models. In this way, any such confounding effects were already statistically accounted for.

Despite birds being negatively associated with *A. phagocytophilum* prevalence, we found no significant relationship of birds with hazard. Birds can occur at high densities, and our results suggest that, at the population level, birds could be increasing *I. ricinus* densities. If birds increase tick densities but decrease *A. phagocytophilum* prevalence, this could result in no overall effect of birds on the environmental hazard of *A. phagocytophilum* (the density of infected ticks). A similar mechanism has been hypothesised for the effect of deer on *B. burgdorferi* s.l.: deer can reduce *B. burgdorferi* s.l. prevalence but, because they have positive effects on tick density, there can be no overall effect of deer on environmental hazard of Lyme disease (Gandy, Kilbride, et al. 2022).

The likelihood of a bird becoming infested with ticks is largely dictated by the foraging, nesting and roosting behaviours unique to each bird species (Heylen 2016; James et al. 2011). Therefore, the contributions of birds to the prevalence and hazard of tick‐borne pathogens are highly species dependent, with ground‐foraging birds such as song thrushes (*Turdus Phylomelos*) and European blackbirds (*T. merula*) being particularly implicated in carrying *I. ricinus* ticks and *B. burgdorferi* s.l. (Heylen 2016; James et al. 2011). European blackbirds and song thrushes were not caught at the 10 sites we used for surveying birds. Instead, the five species of bird included in our analysis were Eurasian chaffinches, Eurasian wrens, European robins, dunnocks, and great tits. These species were the most abundant at our sites and were those found to be carrying *I. ricinus* ticks. They are resident year‐round in the UK, abundant in a range of habitats, and are widely distributed across Europe (Hewson et al. 2007; Walker et al. 2023). These species therefore have the potential to influence *A. phagocytophilum* ecology in many regions and countries. Our results might suggest that conservation measures that increase the abundance of ground‐foraging birds, such as woodland regeneration, may help to reduce the prevalence of *A. phagocytophilum* . However, without a matched reduction in hazard, due to the role these bird species can play in feeding immature *I. ricinus* ticks, an increase in bird abundance may not result in reduced risk to livestock and humans. Increased bird abundance can also be associated with an increase in the circulation of bird‐associated genospecies of *B. burgdorferi* s.l. (Heylen 2016), one of which ( *B. garinii* ) can cause Lyme disease in humans (Baranton et al. 1992; Strle et al. 2006). Therefore, it is possible that environmental conditions (such as abundance of certain hosts) that decrease the prevalence of one pathogen may increase the prevalence of another pathogen. Indeed, previous studies have reported a negative relationship between the prevalences of *A. phagocytophilum* and *B. burgdorferi* s.l. (Gandy, Hansford, et al. 2022; Knoll et al. 2021). Future research could test these relationships to develop a deeper understanding of the role of birds and other hosts in *A. phagocytophilum* ecology. In particular, further research into scenarios with potential trade‐offs between pathogen prevalence and tick production which shape hazard, and trade‐offs between different tick‐borne pathogens, would help to address important gaps in knowledge.

Against our prediction that rodents would, like birds, reduce *A. phagocytophilum* prevalence, we found no evidence of an effect of rodents on prevalence. However, the abundance of rodents was low in many of our sampled sites in our two years of sampling. It remains possible that rodents could be associated with lower *A. phagocytophilum* prevalence in *I. ricinus* , in regions and years with higher rodent abundance. Furthermore, the accuracy of live rodent trapping in reflecting true rodent abundance can vary with ground vegetation type, introducing error in rodent abundance estimates across different habitats (Gandy, Kilbride, et al. 2022). Having said that, our rodent indices did seem reliable enough to reflect the role of rodents in feeding larval *I. ricinus* ticks, thus augmenting the density of nymphs (Cayol et al. 2017; van Duijvendijk et al. 2015), reflected by a significant positive association between rodent abundance and the environmental hazard of *A. phagocytophilum* .

Our study may have important implications for land management, conservation and disease mitigation strategies. Our findings suggest that measures to reduce the density of deer—a commonly used tool to aid habitat recovery and woodland regeneration—may cause a decrease in both the prevalence and hazard of *A. phagocytophilum* , thus reducing disease risk for humans and livestock. The same conservation or deer management tools can result in higher abundance of several bird species, which may further reduce *A. phagocytophilum* prevalence although, as found here, that will not necessarily result in reduced disease hazard due to the role of birds in feeding immature ticks. There is also the possibility that higher bird densities could lead to higher prevalence or hazard of other tick‐borne pathogens which are transmitted by birds, such as *B. garinii* (Heylen 2016).

## Author Contributions

**William McLellan:** data curation (equal), formal analysis (lead), writing – original draft (lead), writing – review and editing (lead). **Sara Gandy:** formal analysis (supporting), supervision (supporting), writing – original draft (supporting), writing – review and editing (supporting). **Jaboury Ghazoul:** funding acquisition (equal), investigation (supporting). **Fanny Olsthoorn:** data curation (supporting), investigation (lead). **Livia May:** investigation (supporting). **Jude Eze:** formal analysis (supporting), supervision (supporting), writing – review and editing (supporting). **Mara Rocchi:** supervision (supporting), writing – review and editing (supporting). **Hein Sprong:** funding acquisition (equal), investigation (lead). **Lucy Gilbert:** conceptualization (lead), formal analysis (supporting), investigation (supporting), supervision (lead), writing – original draft (supporting), writing – review and editing (supporting).

## Funding

Field data collection was funded by ETH Zurich. Data analysis was funded by the Scottish Government Rural and Environment Science and Analytical Services Division (RESAS) project reference MRI‐A2‐10 and by the Moredun Foundation. LG and SLG were supported by the Natural Environment Research Council UK (reference NE/W00299X/1).

## Conflicts of Interest

The authors declare no conflicts of interest.

## Supporting information


**Data S1:** ece374308‐sup‐0001‐supinfo.xlsx.

**Data S2:** ece374308‐sup‐0002‐supinfo.R.

## Data Availability

The data that support the findings of this study are openly available at https://doi.org/10.5061/dryad.v6wwpzh8m.

## Appendix

**TABLE A1:** Sampling details for each site showing the total number of visits, the specific years each site was visited for estimating host abundance indices (deer, sheep, rodents, and birds), and the mean *A. phagocytophilum* prevalence and hazard for each site visit.

Site	Number of visits for tick surveys	Tick, sheep, and deer survey years	Rodent survey years	Bird survey years
BM1	5	2018, 2019, 2020	2018, 2019	2018
BM2	4	2018, 2019		2018
BM3	3	2018		
BM4	6	2018, 2019, 2020	2018, 2019	2018
BM5	3	2018		
BM6	6	2018, 2019, 2020	2018, 2019	2018
BM7	3	2018		
BM8	6	2018, 2019, 2020	2018, 2019	2018
H1	3	2018		
H2	4	2018, 2020	2019	
H3	3	2018		
H4	4	2018, 2020	2019	
H5	3	2018		
H6	3	2018, 2020	2019	
H7	4	2018, 2020	2019	
H8	3	2018		
PM1	3	2018		
PM2	3	2018		
PM3	6	2018, 2019, 2020	2018, 2019	2018
PM4	6	2018, 2019, 2020	2018, 2019	2018
PM5	3	2018		
PM6	4	2018, 2019		2018
PM7	6	2018, 2019, 2020	2018, 2019	2018
PM8	6	2018, 2019, 2020	2018, 2019	2018
PY1	4	2018, 2020	2019	
PY2	4	2018, 2020	2019	
PY3	4	2018, 2020	2019	
PY4	4	2018, 2020	2019	
PY5	3	2018		
PY6	3	2018		
PY7	3	2018		
PY8	3	2018		
SM1	4	2018, 2020	2019	
SM2	4	2018, 2020	2019	
SM3	3	2018		
SM4	4	2018, 2020	2019	
SM5	4	2018, 2020	2019	
SM6	3	2018		
SM7	3	2018		
SM8	3	2018		

*Note:* Data on *Ixodes ricinus* densities and *A. phagocytophilum* prevalence and hazard were gathered during each of the site visits.
